# Predictors of Mean Arterial Pressure Morning Rate of Rise and Power Function in Subjects Undergoing Ambulatory Blood Pressure Recording

**DOI:** 10.1371/journal.pone.0093186

**Published:** 2014-03-25

**Authors:** Geoffrey A. Head, Nick Andrianopoulos, Barry P. McGrath, Catherine A. Martin, Melinda J. Carrington, Elena V. Lukoshkova, Pamela J. Davern, Garry L. Jennings, Christopher M. Reid

**Affiliations:** 1 Baker IDI Heart and Diabetes Institute, Melbourne and Department of Cardiovascular Medicine, Alfred Hospital, Melbourne, Australia; 2 NHMRC Centre for Clinical Research Excellence in Therapeutics, Monash University, Melbourne, Australia; 3 Monash Health Dandenong Australia and Monash University, Melbourne, Australia; 4 Australian Catholic University, Fitzroy, Australia; 5 National Cardiology Research Centre, Moscow, Russia; University of Bologna, Italy

## Abstract

**Background:**

We determined clinical predictors of the rate of rise (RoR) in blood pressure in the morning as well as a novel measure of the power of the BP surge (BP_power_) derived from ambulatory blood pressure recordings.

**Methods:**

BP_power_ and RoR were calculated from 409 ambulatory blood pressure (ABP) recordings from subjects attending a cardiovascular risk clinic. Anthropometric data, blood biochemistry, and history were recorded. The 409 subjects were 20–82 years old (average 57, SD = 13), 46% male, 9% with hypertension but not on medication and 34% on antihypertensive medication.

**Results:**

Average RoR was 11.1 mmHg/hour (SD = 8) and BP_power_ was 273 mmHg^2^/hour (SD = 235). Only cholesterol, low density lipoprotein and body mass index (BMI) were associated with higher BP_power_ and RoR (P<0.05) from 25 variables assessed. BP_power_ was lower in those taking beta-blockers or diuretics. Multivariate analysis identified that only BMI was associated with RoR (4.2% increase/unit BMI, P = 0.020) while cholesterol was the only remaining associated variable with BP_power_ (17.5% increase/mmol/L cholesterol, P = 0.047). A follow up of 213 subjects with repeated ABP after an average 1.8 years identified that baseline cholesterol was the only predictor for an increasing RoR and BP_power_ (P<0.05). 37 patients who commenced statin subsequently had lower BP_power_ whereas 90 age and weight matched controls had similar BP_power_ on follow-up.

**Conclusions:**

Cholesterol is an independent predictor of a greater and more rapid rise in morning BP as well as of further increases over several years. Reduction of cholesterol with statin therapy is very effective in reducing the morning blood pressure surge.

## Introduction

The circadian variation in blood pressure (BP) in humans has been established since the first chronic intra-arterial recordings were made in the late 1960's [1]. It is well known that many factors such as physical activity and periods of rest strongly influence the diurnal BP pattern as well as circadian variation in autonomic and hormonal systems. The importance of this circadian pattern has been brought to the fore by the extensive literature that has been developed over the last two decades to show that cardiovascular events, such as stroke, transient ischemic attacks, myocardial infarction and sudden cardiac death occur most frequently during the morning hours which coincides with the rapid rise in BP and heart rate (HR) [2], [3], [4], [5], [6], [7], [8], [9]. Stroke is up to 3 times more likely in the morning [5], [10] while the Framingham study showed that the incidence of sudden death was nearly twice as likely than at any other time [11]. We have developed a novel mathematical approach to measure the morning surge in BP using a logistic equation that contains separate parameters for the rate of rise (RoR) and the rate of fall as a parameter for the night plateau and the difference between the day and night plateau [12]. The model is therefore able to provide a non-symmetrical fit of any circadian data and can estimate the RoR independent of the rate of fall. When applied to data such as human 24 hour BP and HR recordings, it provides a measure of the RoR of BP and HR in the morning period which is independent of what happens at any other time of the day.

In a prospective study of over 300 subjects, we found that there was a markedly greater RoR in BP and HR in the upper quartile of daytime ambulatory mean arterial pressure (MAP) [13]. This finding was not simply because the underlying BP is higher, as we found no correlation between the RoR in morning systolic BP and the night time systolic BP [13]. Our study showed that the duration of the morning surge is similar between the normotensive and hypertensive participants [13]. Importantly, we have found that the morning RoR is an independent predictor for myocardial infarct and stroke. We have extended this approach and developed a method of assessing the power (effective force) of the morning BP surge (BP_power_) [14]. We found that patients with hypertension and also those with “white coat” hypertension have markedly exaggerated morning surge BP_power_ [14].

Our recent studies suggests that the morning surge is associated with activation of the sympathetic nervous system [15] and that at least in young females, dyslipidaemia is associated with sympathetic activation [16]. Hypertensive patients are often found to have high plasma cholesterol compared to normotensive subjects [17]. Also epidemiological studies show a positive relationship between serum cholesterol and BP levels [18], [19]. The question is whether plasma cholesterol or other measures collected as part of the monitoring of cardiovascular health contribute to the magnitude of the morning BP surge. In a small preliminary study that included only 38 normotensive and 42 hypertensive subjects Martin and colleagues found that there was an association between fasting LDL cholesterol and morning BP surge as assessed by 4 different methods including the RoR and BP_power_ [20]. These associations were independent of age or waist circumference [20] but a covariate analysis was not performed due to the limited numbers. Thus the association between cholesterol and the morning surge needs to be assessed with a much larger study in order to account for cofounding factors. The aim of the current study was to assess the relationship between plasma cholesterol and the morning BP surge (rate and power) in a cross sectional and also a 1–2 year follow up study. We also examined the effect of statin therapy and included other more established methods of calculating the morning BP surge [21].

## Methods

A total of 416 subjects were prospectively recruited from the Healthy Hearts Clinic of Baker IDI Heart and Diabetes Institute or from patients attending the Hypertension Diagnostic Service at the Alfred Hospital Heart Centre (funded and staffed by the Baker IDI Heart and Diabetes Institute) or Monash Health (Dandenong Hospital). Seven subjects were excluded as having nocturnal rising rather than dipping. Of the 409 remainder, 213 were re-examined in follow up after a median 1.8 years (range 1.1 – 2.2). The procedures were approved by The Alfred and Monash Health Human Research Ethics Committee (No: 132/00) and conform to the ethical principles of the Declaration of Helsinki. All subjects gave their written informed consent. Participants fasted overnight and arrived in the morning for clinic assessment of BP, blood sampling for biochemistry, anthropometrics and questionnaires for medical history after which an ABP device was fitted to the non-dominant arm and the patients briefed on the correct use of the device.

### Cardiovascular measurements

ABP was recorded during a typical day using SpaceLabs 90207 or 90217 units (SpaceLabs Medical Inc., Redmond, WA, USA) or Meditech CardioTens (Meditech Ltd, Budapest, Hungary), which were set to measure BP every 30 minutes from late morning for 26 hours. The first and last hour of the recordings were not included in the analysis as they involved fitting and removing the device in the clinic. Diaries were kept by some patients to record daily activities including awake and asleep times. Clinic BP was determined in the reclining position using a mercury sphygmomanometer after 5 minute rest and an average of three readings.

### Analysis of ambulatory curves

ABP recording data were fitted to a 6 parameter double logistic equation as described previously [22]. The principle involves the multiplication of the RoR by the amplitude of the rise which are calculated from our standard six parameter logistic equation [12]. The method is independent of the recorded waking time as we have previously shown these to be unreliable in predicting the peak in BP surge [23]. The novel power function is the first derivative of the logistic curve multiplied by the amplitude which is the day night difference between plateaus [14]. In addition, we included previously used methods of calculating the morning BP surge (MBPS) which were the night minimum minus the post awake 2 hour period (MBPS_NightMin_) and the pre awake minus the post awake 2 hour period (MBPS_pre-awake_) according to the method of Kario and colleagues [21]. Our modification was to use mean BP rather than systolic BP in order to be comparable to the morning BP power.

### Statistical analysis

Data are presented as mean ± standard deviation (SD) of the between-patient variation for continuous variables and frequency (%) for categorical variables. Using the natural log of RoR and BP_power_ as outcomes, baseline univariate predictors were determined using linear regression analysis. The significant univariate predictors were then used in the multivariate analysis. Similarly, for predictors of the change in RoR and BP_power_ as the outcome, the difference between follow up and baseline were used in both the uni- and multi-variate analysis. Slopes were considered significant when P<0.05. Analysis was performed with STATA version 10 Data Analysis and Statistical Software (StataCorp, College Station, Texas, USA.).

## Results

### Subject characteristics

The study group of 409 subjects was made up of 54% female and 46% male subjects with an average age of 57 years (SD = 13, range 20–83, median 58, Table 1). Average body mass index (BMI) was 26.5 kg/m^2^ with plasma total cholesterol of 5.2 mmol/L, low density lipoprotein (LDL) 3.4 mm/L, high density lipoprotein (HDL) 1.4 mmol/L and triglycerides 1.3 mmol/L (Table 1). While males and females were of similar ages and BMI plasma cholesterol including HDL and LDL were slightly higher in females and triglycerides were lower (Table 1, P<0.05 for all). Males also showed elevated daytime, night time MAP as well a lesser day night difference than females but there was no difference between the clinic systolic BP or diastolic BP values (Table 2). Males and females had similar RoR and BP_power_ being on average 11 mmHg/h (SD = 8) and 273 mmHg^2^/h (SD = 235).

**Table 1:** Clinical characteristics of subject groups.

Subjects	Total	Females	Males	P1	Baseline	Follow up	P2
Number	409	222	187		213	213	
Age (years)	56.8±12.8	56.6±12.7	57.1±12.9	0.7	58±12.7	59.9±12.6	0.2
Weight	77.7±14.6	72.9±15	83±12.1	***<0.001***	77.9±14.5	78.3±15.2	0.8
BMI	26.5±3.9	26.4±4.4	26.7±3.3	0.546	26.9±4	27.1±4.1	0.6
Cholesterol (mmol/L)	5.2±0.9	5.4±0.9	5.1±0.9	***0.004***	5.28±0.82	5.03±0.89	***0.013***
LDL (mmol/L)	3.4±0.9	3.5±0.9	3.2±0.9	***0.041***	3.3±0.8	3.01±0.8	***0.004***
HDL (mmol/L)	1.4±0.4	1.5±0.4	1.2±0.3	***<0.001***	1.5±0.4	1.4±0.5	0.9
Triglycerides (mmol/L)	1.3±0.7	1.2±0.6	1.5±0.9	***<0.001***	1.4±0.8	1.4±1	1.0
Fasting glucose (mmol/L)	5.4±1.4	5.2±1.3	5.6±1.4	***0.017***	5.6±1.6	5.3±1.5	0.2
Clinic BP>140/90 mmHg (%)	14.4	13.5	15.5	0.7	12.5	15.8	0.5
Antihypertensive medication (%)	33.5	33.3	33.7	1.0	33.0	45.2	0.2
ACE Inhibitors (%)	13.9	13.1	15.0	0.7	13.0	18.7	0.3
ARB (%)	11.7	10.4	13.4	0.5	14.0	18.1	0.5
Beta blockers (%)	9.3	7.2	11.8	0.3	8.0	12.7	0.3
Calcium channel blockers (%)	10.0	9.5	10.7	0.8	11.5	18.7	0.2
Diuretics (%)	10.3	9.0	11.8	0.5	9.5	15.1	0.3
Statins (%)	18.1	17.6	18.7	0.8	15.5	24.1	0.2

Values are Mean +/- SD, *P*1 is the probability for the comparison between females and males. *P*2 is the probability between baseline and follow up.

**Table 2:** Clinic blood pressures, day and night MAP, day–night difference, morning rate of rise in MAP, and peak morning MAP surge power in subject groups.

Subjects	Total	Females	Males	P1	Baseline	Follow up	P2
Recordings	409	222	187		213	213	
Clinic SBP (mmHg)	137±19	138±21	136±17	0.4	143±21	142±19	0.6
Clinic DBP (mm Hg)	83.8±10.6	83±11	84.8±9.9	0.1	84.9±10.8	84.2±10.9	0.6
Clinic BP>140/90 mmHg (%)	14.4	13.5	15.5		12.5	15.8	
Daytime MAP (mm Hg)	98.2±8.7	97.2±9.6	99.5±7.4	***0.007***	98.4±8.5	98±9.2	0.7
Night MAP (mm Hg)	92.8±8.3	91.4±9.1	94.4±6.9	***<0.001***	93.2±8.2	93.1±8.4	0.9
Day-Night difference (mm Hg)	12.9±6.7	13.5±6.7	12.2±6.5	***0.043***	12.6±6.7	12.1±6.8	0.4
Morning rate of MAP increase (mm Hg/h)	11.1±8.2	11.4±8.2	10.7±8.2	0.4	10.2±8.6	9.0±7.6	0.1
Peak morning surge power (mm Hg2/h)	273±235	289±237	254±231	0.1	248±239	218±222	0.2

Values are Mean +/- SD, *P*1 is the probability for the comparison between females and males. *P*2 is the probability between baseline and follow up.

Based on clinic BP measurements from the entire group, 14% would be considered hypertensive (SBP/DBP>140/90) and 33% were taking antihypertensive therapy with the most common therapy being angiotensin converting enzyme inhibitors at 14%. Based on ABP recordings, 226 (55%) were considered normotensive compared with 183 subjects (45%) with hypertension which included 137 on antihypertensive therapy and 46 not taking therapy.

### Univariate analysis of rate of morning rise in BP and power

**Figure 1:**
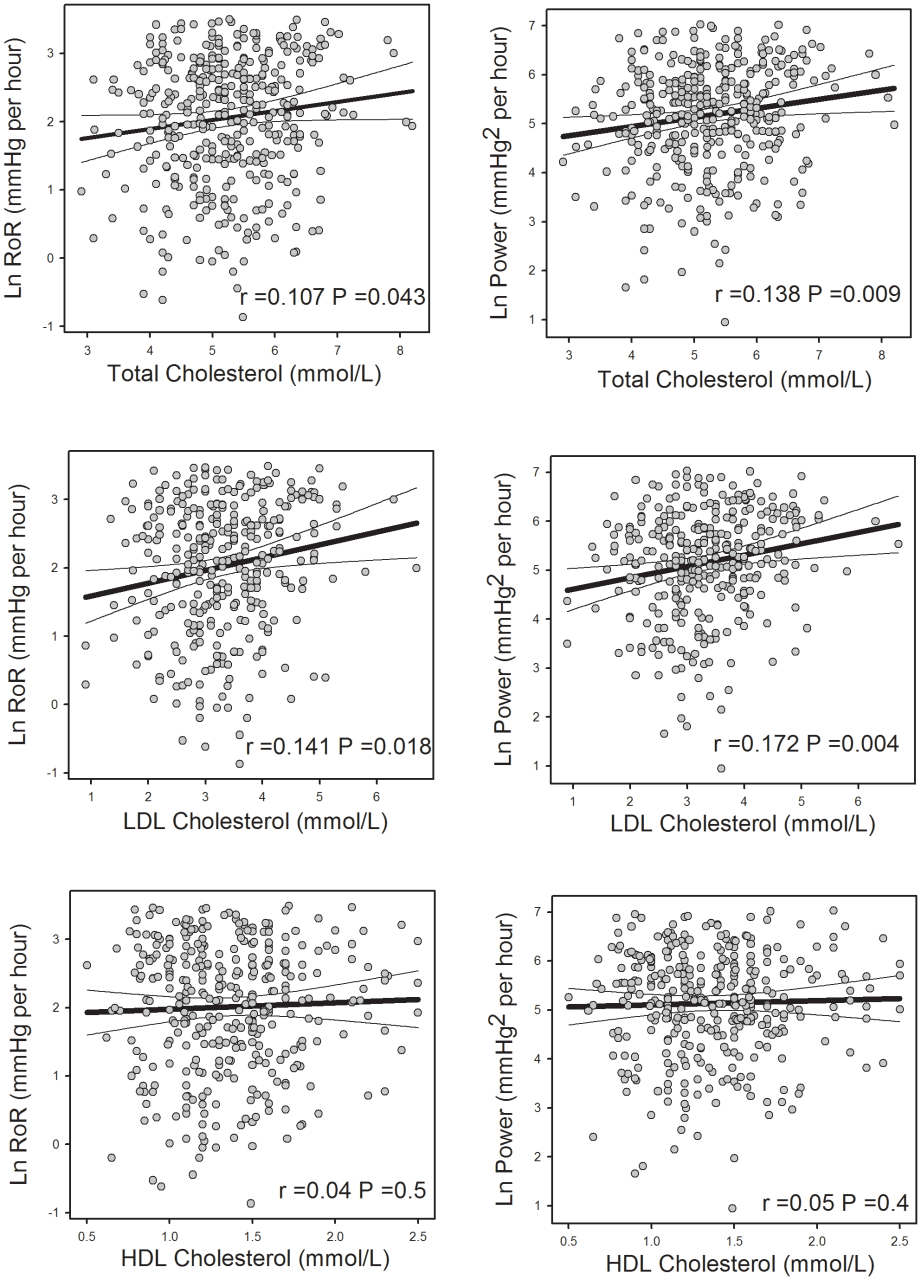
Correlations between natural log morning rate of rise (RoR) in mean arterial pressure (MAP) (left panels) and power (right panels) and plasma levels of cholesterol (top), low density lipoprotein (LDL, middle) and high density lipoprotein (HDL) from subjects (n = 409). Thick line represents least squares regression lines and thin lines are 99% confidence limits. r is the correlation co-efficient and P is the probability.

**Figure 2:**
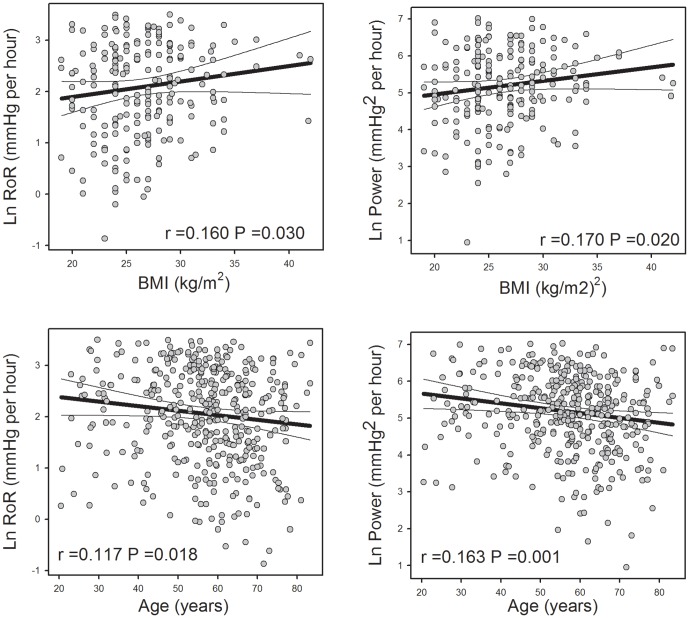
Correlations between natural log morning rate of rise (RoR) in mean arterial pressure (MAP) (left panels) and power (right panels) and body mass index (BMI) and age from subjects (n = 409). Thick line represents least squares regression lines and thin lines are 99% confidence limits. r is the correlation co-efficient and P is the probability.

An initial univariate analysis was performed using the entire 409 recordings. The morning RoR and BP_power_ were first normalised by natural log transformation. Total cholesterol and LDL and BMI but not HDL predicted higher BP_power_ (P<0.05) and RoR (P<0.05) (Table 3, Fig. 1 and Fig. 2). By contrast, a lower BP_power_ and RoR were predicted by age (Fig 2). BP_power_ was proportionally lower in those patients taking beta-blockers or diuretics (Table 3).

**Table 3:** Linear regression coefficient (b), 95% CI and p-value of baseline predictors of the RoR and BP_Power_ as natural log (ln) from a univariate and multivariate analysis of 409 subjects.

Univariate	ln RoR			ln BPPower		
Baseline Predictor	b	95%CI	*P*-value	b	95%CI	*P*-value
Age (yrs)	−0.01	−0.02,−0.001	0.018	−0.01	−0.02,−0.005	0.001
BMI (kg/m^2^)	0.04	0.004,0.07	0.030	0.03	−0.005,0.07	0.087
Male (%)	−0.06	−0.25,0.12	0.488	−0.11	−0.32,0.09	0.282
Clinical systolic HPT (%)	−0.01	−0.21,0.19	0.921	0.06	−0.17,0.29	0.603
Clinical diastolic HPT (%)	−0.12	−0.35,0.11	0.307	−0.13	−0.40,0.13	0.319
BP >140/90 mmHg (%)	−0.10	−0.36,0.16	0.432	−0.10	−0.40,0.19	0.494
Baseline hypertension (%)	−0.10	−0.29,0.09	0.299	−0.09	−0.31,0.13	0.418
Cholesterol (mmol/L)	0.11	0.003,0.22	0.043	0.16	0.04,0.28	0.009
LDL (mmol/L)	0.15	0.03,0.27	0.018	0.20	0.07,0.34	0.004
HDL (mmol/L)	0.09	−0.20,0.37	0.552	0.14	−0.18,0.46	0.402
Triglycerides (mmol/L)	−0.04	−0.17,0.09	0.531	−0.03	−0.18,0.12	0.675
Fasting glucose (mmol/L)	−0.02	−0.09,0.05	0.599	−0.04	−0.12,0.05	0.389
Current Smokers (%)	0.09	−0.38,0.56	0.711	0.04	−0.49,0.58	0.882
Family history of CHD (%)	0.16	−0.07,0.40	0.177	0.25	−0.02,0.51	0.067
Diabetic (%)	−0.15	−0.59,0.30	0.515	−0.13	−0.63,0.37	0.610
Any alcohol (%)	0.05	−0.05,0.15	0.304	0.07	−0.04,0.18	0.219
Antihypertensive medication (%)	−0.14	−0.33,0.05	0.144	−0.13	−0.34,0.09	0.247
ACE Inhibitors (%)	−0.04	−0.29,0.22	0.761	−0.04	−0.33,0.25	0.772
ARB (%)	−0.12	−0.40,0.15	0.378	−0.01	−0.33.0.30	0.938
Beta blockers (%)	−0.30	−0.62,0.01	0.060	−0.49	−0.85,−0.14	0.007
Calcium channel blocker (%)	−0.18	−0.48,0.11	0.221	−0.23	−0.56,0.11	0.186
Diuretics (%)	−0.25	−0.54,0.04	0.094	−0.37	−0.70,−0.04	0.029
Statins (%)	−0.02	−0.25,0.22	0.888	−0.06	−0.33,0.20	0.635
Diabetic medication (%)	−0.03	−0.64,0.60	0.948	−0.10	−0.80,0.61	0.789

Multivariate	Ln RoR			Ln BPPower		
Baseline Predictor	b	95%CI	p-value	b	95%CI	p-value
Age (yrs)	−0.01	−0.2,0.006	0.405	−0.01	−0.02,0.002	0.113
BMI (kg/m^2^)	0.04	0.01,0.08	0.020	-	-	-
Cholesterol (mmol/L)	0.07	−0.09,0.23	0.379	0.16	0.003,0.32	0.047
Beta Blocker	-	-	-	−0.23	−0.71,0.24	0.336
Diuretic	-	-	-	−0.23	−0.71,0.24	0.337

**Figure 3:**
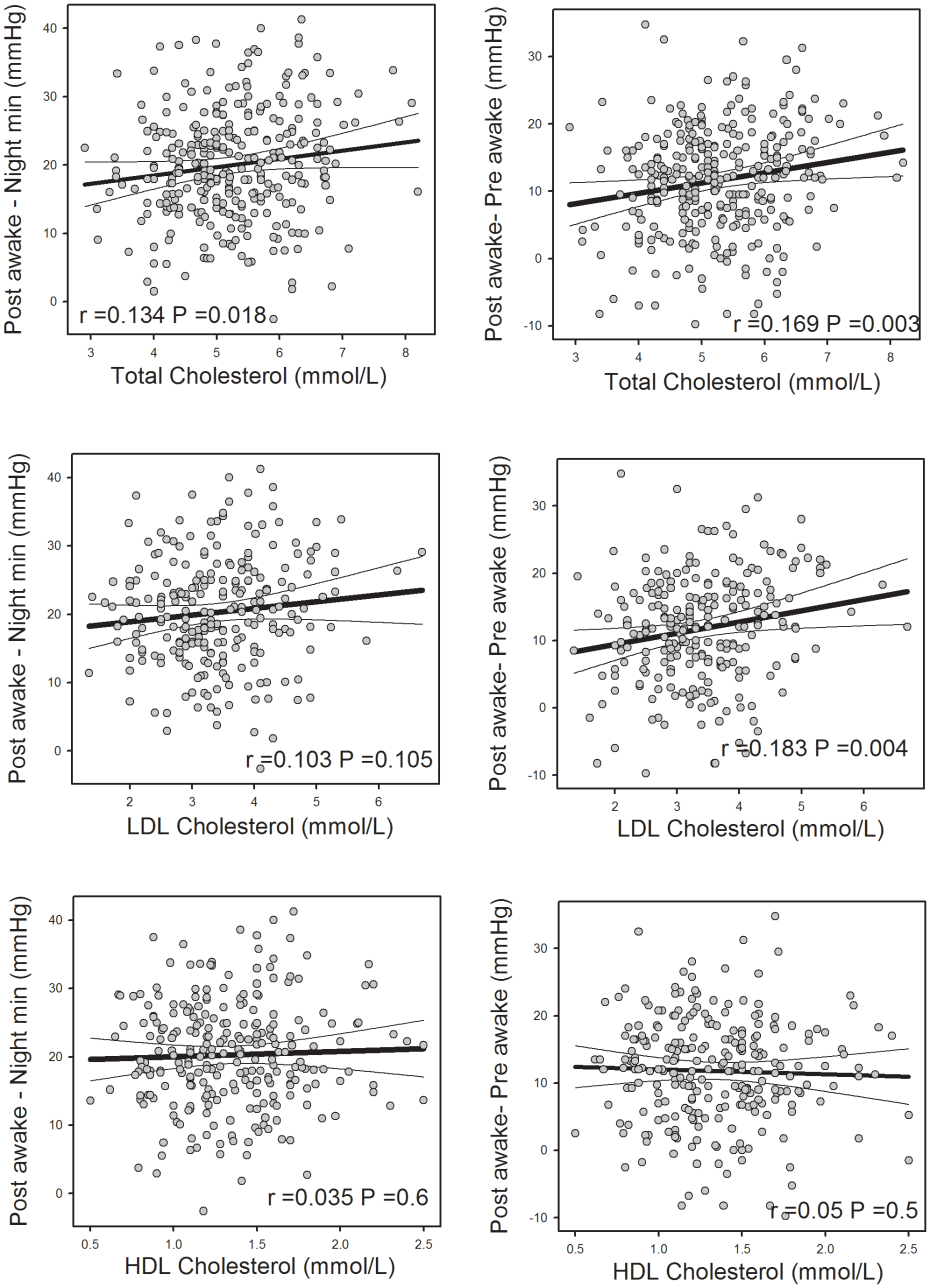
Correlations between post awake 2(MAP) (left panels) and post awake 2 hours minus pre awake 2 hours (right panels) and plasma levels of cholesterol (top), low density lipoprotein (LDL, middle) and high density lipoprotein (HDL) from subjects (n = 307). Thick line represents least squares regression lines and thin lines are 99% confidence limits. r is the correlation co-efficient and P is the probability.

We also found there was an association of MBPS_NightMin_ and MBPS_pre-awake_ with cholesterol (P<0.05), MBPS_pre-awake_ was associated with LDL cholesterol but neither were associated with HDL (Fig. 3).

### Multivariate analysis of rate of morning rise in BP and power

Multivariate analysis determined that only BMI predicted morning RoR (4.2% increase in per unit BMI, P = 0.020), while total cholesterol was the only remaining predictor for BP_power_ (17.5% increase/mmol/L, P = 0.047, Table 3).

### Predicting change in analysis of rate of morning rise in BP and power

A total of 213 subjects from the main study underwent follow up ABP monitoring (median follow-up time of 1.8 years) from whom we calculated the predictors of a change in RoR and BP_power_ between baseline and analyzed this by univariate and multivariate covariance. The univariate analysis indicated that baseline total cholesterol, LDL, triglycerides, fasting glucose and diabetic medication were predictors of increases in RoR and BP_power_ (Table 4, P<0.05). However, multivariate analysis showed after adjustment for baseline level of RoR and BP_power_ as well as time between visits, that only baseline total cholesterol was a predictor for increasing RoR and BP_power_ (P<0.05, Table 4). No association was found with 24 hour MAP or statin use in any of the analyses.

**Table 4:** Linear regression coefficient (b), 95% CI and p-value of baseline predictors of the change in natural log (ln) RoR and BP_power_ between first follow-up and baseline visits from a univariate and multivariate ANCOVA analysis of recordings from 213 subjects.

ANCOVA - Univariate	Δ ln RoR#			Δ ln BPPower∧		
Baseline Predictor	b	95%CI	*P*-value	b	95%CI	*P*-value
Cholesterol (mmol/L)	0.21	0.05,0.37	0.009	0.29	0.11,0.48	0.002
LDL (mmol/L)	0.24	0.05,0.44	0.015	0.32	0.09,0.56	0.008
Triglycerides (mmol/L)	0.23	0.05,0.42	0.012	0.31	0.10,0.52	0.004
Fasting glucose (mmol/L)	-0.11	-0.20,-0.01	0.024	-0.12	-0.23,-0.02	0.025
Diabetic medication	-0.95	-1.71,-0.18	0.015	-1.49	-2.38,-0.60	0.001

ANCOVA -Multivariate	Δ ln RoR#			Δ ln BPPower∧		
Baseline Predictor	b	95%CI	*P*-value	b	95%CI	*P*-value
Cholesterol (mmol/L)	0.21	0.05,0.37	0.012	0.29	0.11,0.48	0.002
Fasting glucose (mmol/L)	-0.08	-0.18,0.01	0.091	-0.09	-0.20,0.02	0.108

# Also adjusted for baseline ln ROR and time between visits,

∧ Also adjusted for baseline ln BP_power_ and time between visits.

### Effect of starting statin therapy

Statin therapy was commenced between the initial and subsequent ABP assessments in 37 subjects (Table S1). BP_power_ was reduced by 46% but not the day night difference (−15%) or the morning RoR (−26%). Also the MBPS was not affected by the commencement of statin therapy. The only other change was an increase in the use of angiotensin receptor blockers but we have previously shown that the use of these agents does not affect morning BP_power_. Stain therapy reduced plasma cholesterol by 1.2 mmol/L due to a reduction in LDL but not HDL. Plasma triglycerides were also reduced by 35%.

No difference in any parameter was observed in an aged, weight and BMI matched control group (n = 100) that had not started statin therapy by the follow up study. The baseline characteristics were identical for anthropomorphic, plasma lipids and antihypertensive treatment except that triglycerides were lower.

## Discussion

The current study determined independent predictors of the morning surge in BP using a novel method of analysis of the RoR as well as an index of the “power” of the BP surge derived from the product of the rate and amplitude [12], [13]. We examined over 20 clinical anthropomorphic, biochemical and treatment indices to find that the only independent predictor of the morning BP_power_ was plasma cholesterol and the best predictor of morning RoR was BMI. Importantly, we also found that plasma cholesterol at baseline was the only independent predictor of an increase in the RoR and an increase in the morning BP_power_ over the 1–2 year follow up examination. The latter analysis was adjusted for baseline levels of BP_power_ which eliminates the cross-sectional association between cholesterol and morning power from the follow up study. We also found that commencing statin therapy markedly reduced the BP_power_ while no change was observed in those that did not commence a statin between visits. These findings suggest that plasma cholesterol may be intimately linked with the underlying pathological process that is leading to a greater morning BP surge rather than a simple association.

The mechanism involved in the increase in BP and HR in the morning period involves activation of a number of systems associated with arousal as well as circadian rhythms associated with transitioning from dark to light. The transition from sleep to awake is associated with activation of the sympathetic nervous system and increasing levels of plasma catecholamines leading to greater sympathetic vasoconstrictor tone [24]. Also during the morning period compared with evening, sympathetic baroreflex gain is reduced [25], plasma cortisol is elevated [26] and endothelium dependent forearm vasodilatation is reduced [26]. By contrast there is little circadian rhythm in plasma cholesterol [26] suggesting that the link between cholesterol and the morning BP_power_ in both the cross sectional and longitudinal study is not simply a co-incidence of an association of circadian rhythms. Furthermore, we also observed significant correlations with previous methods of determining the morning BP surge and cholesterol and LDL cholesterol. These methods developed by Kario and colleagues encapsulate the change in BP pre and post waking or post awake minus night minimum. The latter essentially indicates the magnitude of the morning rise [21], [27], [28].

Activation of the sympathetic vasomotor drive associated with arousal in the morning period is a particularly important mechanism influencing the rise in BP. We have recently found that there was a close association between an acute increase in muscle sympathetic activation due to a cold pressor test and the morning BP_power_ [15]. Thus it would appear that there is a major contribution of the morning surge in BP and hence BP_power_ from the sympathetic nervous system. We have recently published that dyslipidaemia (high cholesterol) in young females is associated with high levels of sympathetic activity compared to normal as measured by micro-neurography [16]. This association was independent of age, height, weight or BMI. Furthermore Ekstedt and colleagues found that total cholesterol, LDL and cortisol but not HDL, triglycerides or insulin were associated with the frequency of micro-arousals during sleep [29]. This suggests that inappropriate activation of the sympathetic nervous system during sleep (micro-arousals) and at the end of sleep (morning power) can be predicted by elevated cholesterol.

Plasma cholesterol has long been associated with elevated BP particularly from major epidemiological studies [17], [18], [19]. Recent reports using the hypercholesterolemic LDL receptor knockout mouse suggest that a possible mechanism may involve cerebral oxidative stress [30], [31], [32]. These mice have lower mitochondrial oxidative capacity due to greater consumption of NADPH-linked substrates [31] and display greater sympathetic vasomotor drive [30]. We suggest that this mechanism may underlie the association between the morning BP_power_ and cholesterol via a greater sympathetic contribution to the morning arousal surge in BP. In support, studies have previously shown an association between oxidative stress and sympathetic contributions to obesity induced hypertension [33], renovascular hypertension [34], [35], salt sensitive hypertension [36], a mouse model of neurogenic hypertension [37]. Conversely inhibiting oxidative stress leads to sympatho-inhibition [38]. An additional mechanism may involve the known effect of LDL cholesterol to increase the expression of angiotensin type 1 receptors in the vasculature [39], [40] and promote the development of hypertension [41]. Activation of the renin-angiotensin system by hypercholesterolemia may further activate the sympathetic nervous system through a variety of mechanisms [42].

The strength of our study is that the analysis has used a large data set with follow up assessments allowing for within subject analysis which complements the cross sectional analysis. The multivariate analysis model has allowed us to account for significant factors such as types of treatment. Interestingly, beta blockers and diuretics were negatively correlated to morning BP_power_ but none of these treatments were independent of cholesterol. We had previously found that patients taking diuretics had reduced BP_power_ [14] and those taking beta blockers trended similarly but did not reach statistical significance [14]. We did not observe in the cross sectional study an association between statin treatment and morning power since only those patients with high cholesterol levels would be given statins. When we compared the same subjects before and after commencing statin therapy, a clear reduction in morning BP_power_ was observed. The reduction cannot be explained by any other recorded parameter and was not observed in an age, weight and BMI matched group that did not begin statin therapy over a similar time frame.

In conclusion, we used a new measure of the morning BP surge which incorporates the RoR in BP multiplied by the amplitude, namely BP_power_ to show that in a large well characterized group of subjects that plasma cholesterol was the sole independent correlate. Importantly cholesterol was the sole predictor of a long term increase in morning BP_power_ after adjustment for baseline BP_power_. We suggest that the mechanism may relate to increased cerebral oxidative stress or activation of the renin-angiotensin system leading to an activation of the sympathetic nervous system. Importantly, we found from a limited number of subjects who had commenced statin therapy that reducing cholesterol had a marked effect in reducing BP_power_. These studies suggest an important link between two major risk factors in cardiovascular disease, namely hypertension and dyslipidaemia and offer new insights into their interaction within the central nervous system. BP_power_ may therefore be a useful measure to highlight those subjects at greatest risk of cardiovascular events and for determining the most benefit of antihypertensive and cholesterol lowering therapy.

## Supporting Information

Table S1 **Characteristics and morning surge parameters of 37 subjects before (baseline) and after commencing chronic statin therapy and of 90 age, weight and BMI matched subjects before (baseline) and remaining without statin treatment on follow up.** Values are Mean ± SD. P is the probability for the comparison between baseline and subsequent visit.
(DOC)
