# Supplementary material for: Predictors of Mean Arterial Pressure Morning Rate of Rise and Power Function in Subjects Undergoing Ambulatory Blood Pressure Recording
Source: PLoS One. 2014 Mar 25;9(3):e93186. doi: 10.1371/journal.pone.0093186 (PMC3965554; doi:10.1371/journal.pone.0093186)
Supplement: Table S1 — Characteristics and morning surge parameters of 37 subjects before (baseline) and after commencing chronic statin therapy and of 90 age, weight and BMI matched subjects before (baseline) and remaining without statin treatment on follow up. Values are Mean ± SD. P is the probability for the comparison between baseline and subsequent visit. (DOC) [file pone.0093186.s001.doc]

| **Subjects** | **No Statin Baseline** | **Statin on follow up** | **P1** | **No Statin Baseline** | **No statin on Follow up** | **P2** |
| --- | --- | --- | --- | --- | --- | --- |
| **Number** | **37** | **37** |  | **90** | **90** |  |
| Age (years) | 62.1 ± 12.4 | 65.6 ± 12.3 |  | 62.1 ± 8.8 | 65.1 ± 8.6 |  |
| Weight | 76.6 ± 16.3 | 74.2 ± 13.7 | 0.6 | 76.3 ± 16.7 | 76 ± 14.3 | 0.9 |
| BMI | 27.1 ± 3.6 | 26.7 ± 3 | ***<0.001*** | 27.1 ± 3.9 | 26.6 ± 3.6 | 0.5 |
| Cholesterol (mmol/L) | 5.3 ± 0.6 | 4.1 ± 0.7 | ***<0.001*** | 5.4 ± 0.72 | 5.32 ± 0.9 | 0.8 |
| LDL (mmol/L) | 3.1 ± 0.6 | 2 ± 0.6 | ***<0.001*** | 3.3 ± 0.6 | 3.26 ± 0.8 | 0.7 |
| HDL (mmol/L) | 1.4 ± 0.4 | 1.5 ± 0.5 | 0.3 | 1.5 ± 0.4 | 1.5 ± 0.5 | 0.5 |
| Triglycerides (mmol/L) | 2 ± 1.3 | 1.3 ± 0.8 | ***0.016*** | 1.4 ± 0.9 | 1.4 ± 0.8 | 0.8 |
| Fasting glucose (mmol/L) | 5.5 ± 1.7 | 5.8 ± 1.2 | 0.6 | 5.2 ± 0.7 | 5.2 ± 0.7 | 0.9 |
| Antihypertensive medication (%) | 59.5 | 78.4 | 0.1 | 37.8 | 45.6 | 0.4 |
| ACE Inhibitors (%) | 24.3 | 24.3 | 1.0 | 10.0 | 11.1 | 0.8 |
| ARB (%) | 16.2 | 37.8 | ***0.003*** | 18.9 | 25.6 | 0.3 |
| Beta blockers (%) | 18.9 | 24.3 | 0.4 | 8.9 | 8.9 | 1.0 |
| Calcium channel blockers (%) | 35.1 | 40.5 | 0.5 | 13.3 | 17.8 | 0.4 |
| Diuretics (%) | 16.2 | 21.6 | 0.4 | 11.1 | 17.8 | 0.2 |
| Statins (%) | 0.0 | 100.0 |  | 0.0 | 0.0 |  |
| Daytime MAP (mm Hg) | 99.5 ± 8.2 | 95.4 ± 10.5 | 0.1 | 99.9 ± 9 | 97.7 ± 9 | 0.2 |
| Night MAP (mm Hg) | 95.1 ± 6.9 | 91.8 ± 9.6 | 0.1 | 94.6 ± 8.1 | 93 ± 7.9 | 0.2 |
| Day-Night difference (mm Hg) | 11.2 ± 8.3 | 9.5 ± 7.2 | 0.4 | 13.2 ± 7.2 | 12.1 ± 7.6 | 0.4 |
| Morning rate of MAP increase (mm Hg/h) | 10.3 ± 10.3 | 7.6 ± 5.5 | 0.2 | 9.6 ± 7.8 | 9.2 ± 8.8 | 0.8 |
| BP Power (mm Hg2/h) | 274 ± 353 | 150 ± 128 | ***0.049*** | 241 ± 209 | 234 ± 282 | 0.9 |
| MBPS (mm Hg) | 23.7 ± 13 | 19 ± 9 | 0.1 | 20.9 ± 8 | 21.6 ± 11 | 0.7 |
